# Supplementary material for: Time-Series Chlorophyll Fluorescence Imaging Reveals Dynamic Photosynthetic Fingerprints of sos Mutants to Drought Stress
Source: Sensors (Basel). 2019 Jun 12;19(12):2649. doi: 10.3390/s19122649 (PMC6631407; doi:10.3390/s19122649)
Supplement: Supplementary file 1 [file sensors-19-02649-s001.zip › Additional file 1.pdf]

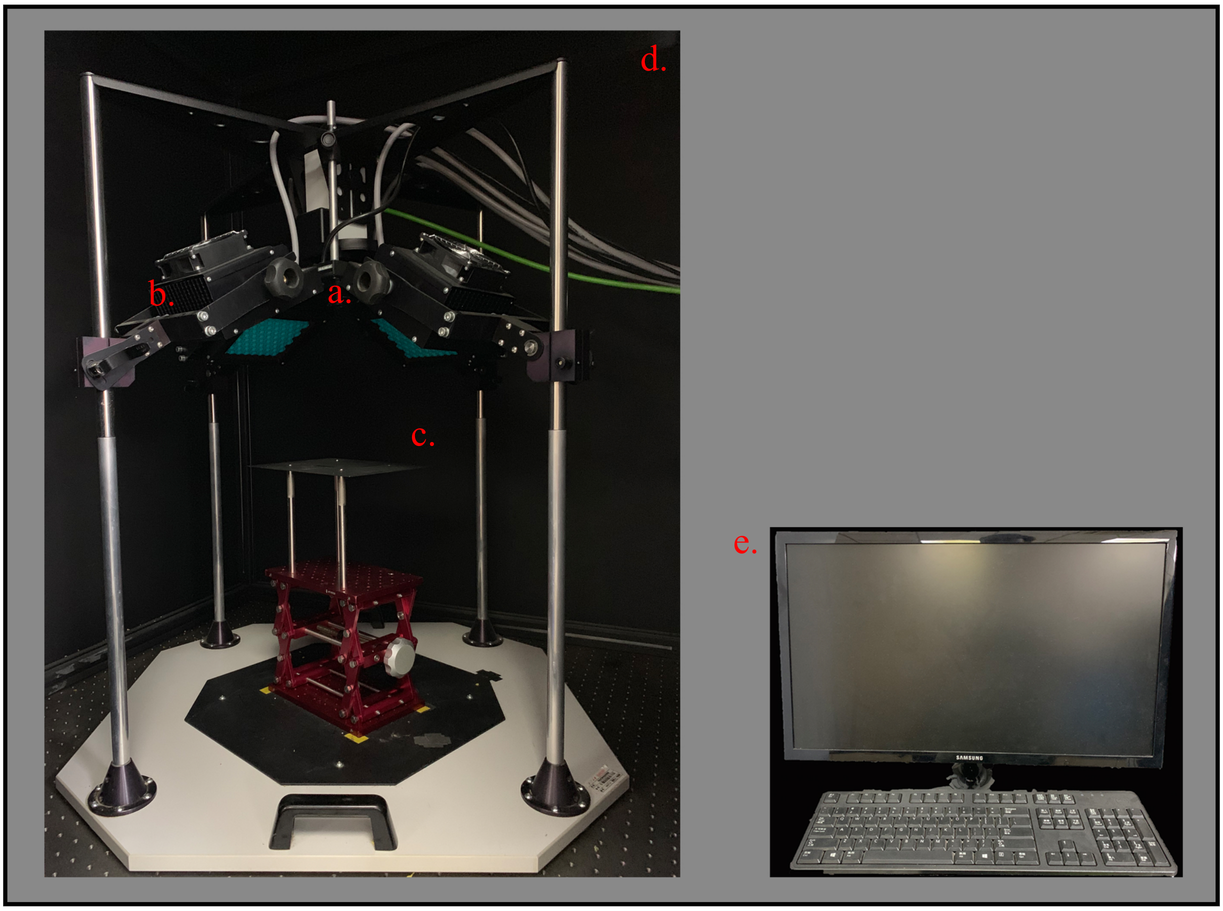

Fig. S1: The detailed setting of ChlF imaging system

a. charge-coupled device (CCD) camera with a prime lens (SV-H 1.4/6, VS Technology, Tokyo, Japan); b. Light emitting diode (LED) panels (45º incident angle); c. Adjustable sample-loading platform; d. Dark environment; e. controling and monitoring computer.
