# Supplementary material for: Time-Series Chlorophyll Fluorescence Imaging Reveals Dynamic Photosynthetic Fingerprints of sos Mutants to Drought Stress
Source: Sensors (Basel). 2019 Jun 12;19(12):2649. doi: 10.3390/s19122649 (PMC6631407; doi:10.3390/s19122649)
Supplement: Supplementary file 1 [file sensors-19-02649-s001.zip › Additional file 2.pdf]

The detailed quenching protocol with major parameter abbreviations and important calculation formulas

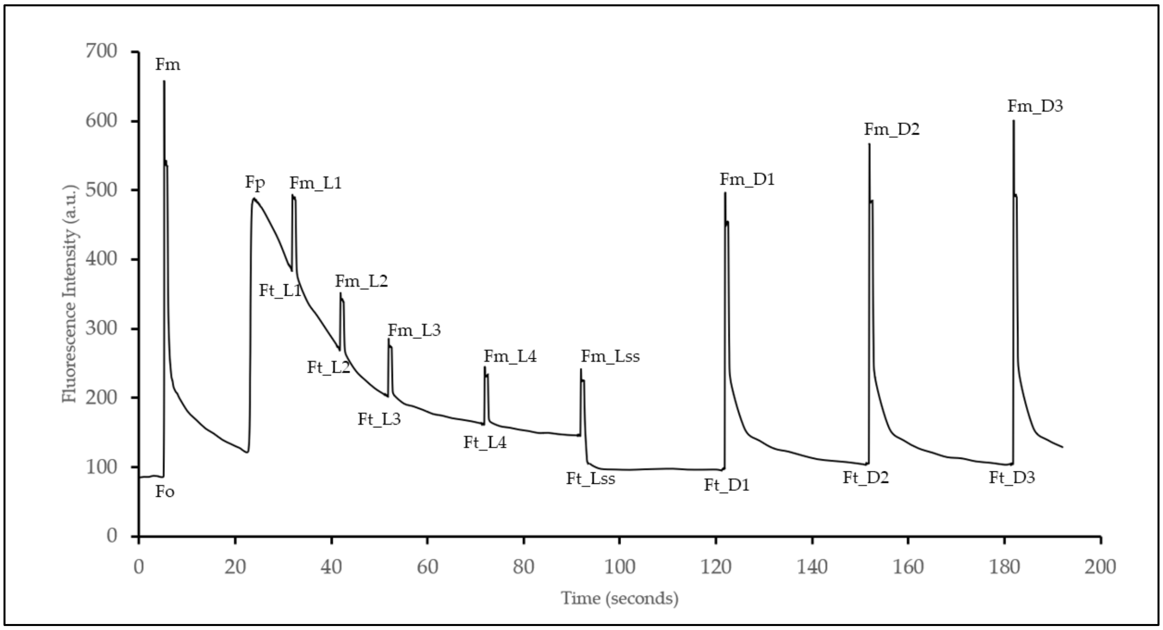

Fig a. Detailed quenching protocols

Abbreviations:

Fo, the minimum fluorescence; *Fm*, maximum fluorescence; *Fm_Ln*, the maximum fluorescence in light; *Fm_Lss*, the steadystate maximum fluorescence in light; *Fm_Dn*, the instantaneous maximum fluorescence signals in dark; *Fp*, the peak rise fluorescence; *Ft_Ln*, the instantaneous fluorescence in light; *Ft_Lss*, the steady-state fluorescence in light; *Ft_Dn,* the instantaneous fluorescence signals before the saturating flashes; *Fv_Lss,* variable fluorescence in the steady state; *Fv_Ln,* variable fluorescence during light adaptation; *Fv'/Fm'*, the trapping efficiency of PSII; *Fs/Fo,* steady-state of chlorophyll fluorescence yield normalized to the minimal fluorescence yield; *NPQ,* instantaneous non-photochemical quenching relaxation; *Fv/Fm, (Fm – Fo) /Fm,* the maximum PSII quantum yield; *NPQ_Ln, (Fm -Fm_Ln)/Fm_Ln,* instantaneous non-photochemical quenching in light; *NPQ_Lss, (Fm -Fm_Lss)/Fm_Lss,* steady-state non-photochemical quenching; *qL, (Fq/Fv)/(Fo/Ft),* coefficient of photochemical quenching measured the fraction of open PSII centers based on a 'lake' model for PSII; *qP, (Fm-Ft)/(Fm-Fo),* coefficient of photochemical quenching; *QY, (Fm-Ft)/Fm,* instantaneous PSII quantum yield; *Rfd_Ln, (Fp-Ft_Ln)/Ft_Ln,* instantaneous fluorescence decline ratio in light; *qN_Dn, (Fm-Fm_Dn)/(Fm-Fo_Dn),* coefficient of non-photochemical quenching during dark relaxation; *qN_Ln, (Fm-Fm_Ln)/ (Fm-Fo_Ln),* coefficient of non-photochemical quenching during light adaptation; *qL_Ln, (Fm_Ln-Ft_Ln)/(Fm_Ln-Fo_Ln),* coefficient of photochemical quenching during light adaptation; *qL_Lss, (Fm_Lss-Ft_Lss)/(Fm_Lss-Fo_Lss),* coefficient of photochemical quenching in steady-state; *ΦPSII_Lss, (Fm_Lss - Ft_Lss)/Fm_Lss,* steady-state PSII quantum yield; *ΦPSII_Ln, (Fm_Ln - Ft_Ln) /Fm_Ln,* PSII quantum yield in light.
