# Supplementary material for: Time-Series Chlorophyll Fluorescence Imaging Reveals Dynamic Photosynthetic Fingerprints of sos Mutants to Drought Stress
Source: Sensors (Basel). 2019 Jun 12;19(12):2649. doi: 10.3390/s19122649 (PMC6631407; doi:10.3390/s19122649)
Supplement: Supplementary file 1 [file sensors-19-02649-s001.zip › Additional file 4R.pdf]

Additional file 4: File S2 Detailed SAEs computing environment and procedures

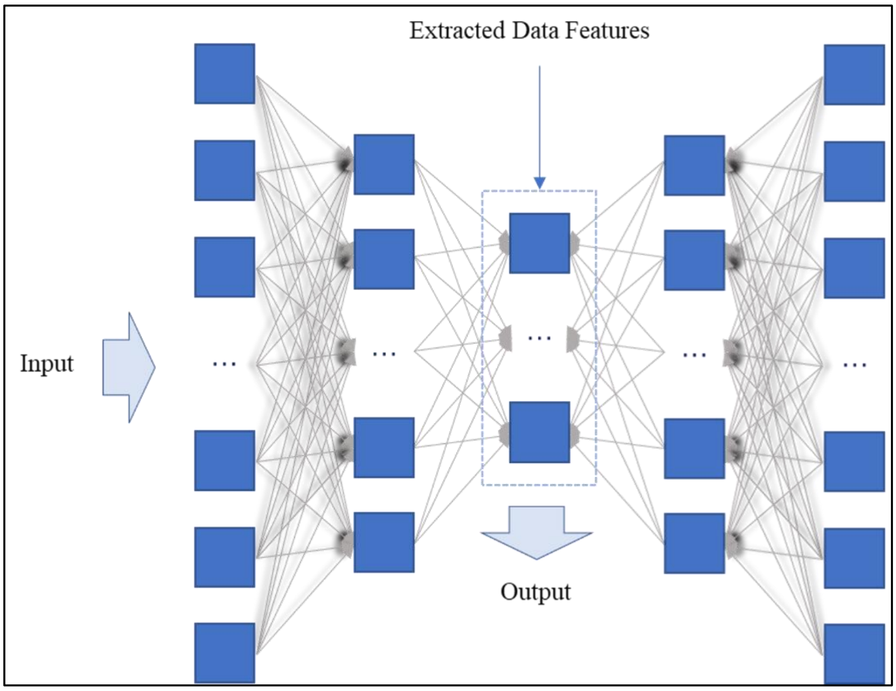

Fig a. The network architecture of SAEs

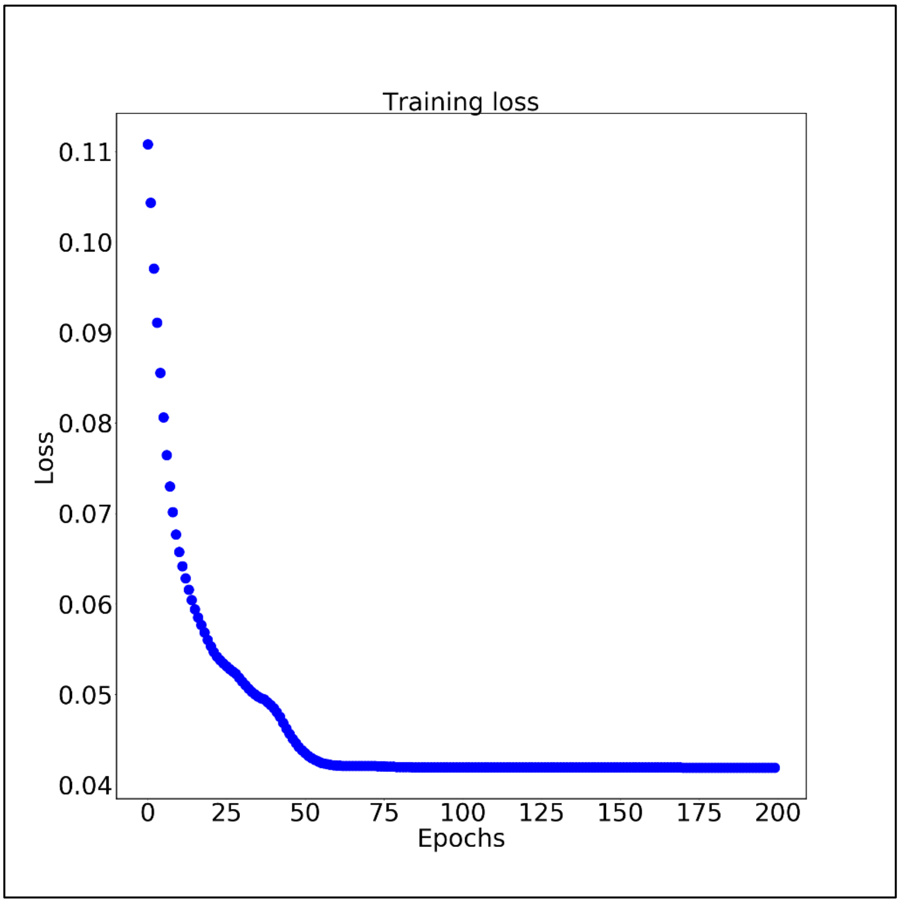

Fig b. Training loss over 200 epochs

Sparse Auto Encoders (SAEs) was employed to analysis ChlF data over time to extract features of different genotypes. The aim of SAEs is to extract the latent or compressed feature of the input, which is achieved by minimizing the reconstruction errors between original data and the reconstructed data using the extracted features as representatives. SAEs was performed on the python program (Python 3.6) based on high-level neural networks API Keras (https://keras.io/) in PyCharm IDE (PyCharm 2018.2.2) for Microsoft Windows professional. All of the encoded and decoded activation function was achieved through Relu which is widely used in the deep learning algorithms training. The training was conducted on a Dell workstation with two Intel® Xeon(R) CPU E5-2620 v4 @ 2.10 GHz, 32.0 GB RAM, and one NVIDIA GeForce GTX 1080 Ti.

The construction of the SAEs can be basically divided into input layer, several hidden layers and one output layer (Fig a). The hidden layer represents the key features extracted by the encoding layers from the input vector, and its reliability is validated by back propagating the decoding layers. The training epochs for each sample was set to 200. Based on the training loss over epochs, most of the sample training loss become stable around 0.04 after 75 times training (Fig b). Therefore, 200 times training epochs are enough for the SAEs neural network of this study. The SAEs applies an auto-encoder which could map x_i of the training data matrix $X= X_i \in R^{\wedge n}$ from N subjects to a latent representation $yi \in R^o$ (o represents output, which is the extracted vector features) through a linear deterministic mapping and a nonlinear activation function. Therefore, in this study, the data of all parameters throughout the whole experimental period (with 98 variables in total) was inputted as vectors in the shape of (1×98). After down sampling, the hidden layers vectors were reconstructed in the shape of (1×60), (1×40), (1×20), (1×10) and (1×5), respectively. The decoding layers then reconstructed the original vector (1,98) from the final vector (1,5) in a reverse way with feedback propagation to validate the extracted features. The selected vector features were encoded as (1,5) for machine learning classification.
